# Return on investment in science: twenty years of European Commission funded research in Alzheimer’s dementia, breast cancer and prostate cancer

**DOI:** 10.1186/s12962-024-00540-5

**Published:** 2024-06-16

**Authors:** Mihajlo Jakovljevic, Pierre Deceuninck, Francesca Pistollato, Evangelos Daskalopoulos, Camilla Bernasconi, Florabela Carausu, Matilde Rosa, Artemis Progri, Martina Makarieva, Kristijan Krstic

**Affiliations:** 1 UNESCO - The World Academy of Sciences (TWAS), Trieste, Italy; 2 Shaanxi University of Technology, Hantai District, Hanzhong, 723099 Shaanxi China; 3 Department of Global Health Economics and Policy, University of Kragujevac, Kragujevac, Serbia; 4 European Commission, Joint Research Centre (JRC), Ispra, Italy; 5 Humane Society International, Europe, Av. Des Arts 50, 1000 Bruxelles, Belgium; 6 GOPA Worldwide Consultants GmbH, Hindenburgring 18, 61348 Bad Homburg Vor Der Höhe, Germany; 7 GOPA Luxembourg, Bereldange, Luxembourg; 8 Social Data Lab, Lisbon, Portugal; 9 Clinic of Physiatrics and Rehabilitation Medicine, University Clinical Centre Kragujevac, Kragujevac, Serbia

**Keywords:** European Commission, Alzheimer’s disease, Breast cancer, Prostate cancer, Indicators, Funding, Investment, Outcomes, Policy, Innovation, Pharmaceuticals, Medical care, FP5, FP6, FP7, Horizon 2020, Return on investment (RoI), Public P&R funding, Research policy, Public investment

## Abstract

Alzheimer’s disease (AD), breast cancer (BC) and prostate cancer (PC) continue to be high in the research and innovation agenda of the European Commission (EC). This is due to their exceptionally large burden to the national health systems, the profound economic effects of opportunity costs attributable to decreased working ability, premature mortality and the ever-increasing demand for both hospital and home-based medical care. Over the last two decades, the EC has been steadily increasing both the number of proposals being funded and the amounts of financial resources being allocated to these fields of research. This trend has continued throughout four consecutive science funding cycles, namely framework programme (FP)5, FP6, FP7 and Horizon 2020 (H2020). We performed a retrospective assessment of the outputs and outcomes of EC funding in AD, BC and PC research over the 1999–2019 period by means of selected indicators. These indicators were assessed for their ability to screen the past, present and future for an array of causal relationships and long-term trends in clinical, epidemiological and public health sphere, while considering also the broader socioeconomic impact of funded research on the society at large. This analysis shows that public–private partnerships with large industry and university-based consortia have led to some of the most impactful proposals being funded over the analysed time period. New pharmaceuticals, small molecules and monoclonal antibodies alike, along with screening and prevention, have been the most prominent sources of innovation in BC and PC, extending patients’ survival and enhancing their quality of life. Unlike oncology, dementia drug development has been way less successful, with only minor improvements related to the quality of supportive medical care for symptoms and more sensitive diagnostics, without any ground-breaking disease-modifying treatment(s). Significant progresses in imaging diagnostics and nanotechnology have been largely driven by the participation of medical device industry multinational companies. Clinical trials funded by the EC were conducted, leading to the development of brand-new drug molecules featuring novel mechanisms of action. Some prominent cases of breakthrough discoveries serve as evidence for the European capability to generate cutting-edge technological innovation in biomedicine. Less productive areas of research may be reconsidered as priorities when shaping the new agenda for forthcoming science funding programmes.

## Introduction

The European Commission (EC) has funded four consecutive science funding cycles called Framework Programmes FP5, FP6, FP7 and Horizon 2020 (H2020) over the last two decades. A vast amount of this research financing was dedicated to research on Alzheimer’s disease (AD), breast cancer (BC) and prostate cancer (PC). There is a growing body of evidence that neurodegenerative disease and cancer present an overwhelming burden to the European Union (EU) societies. Several underlying factors make these particular clinical entities unique among the other non-communicable chronic diseases (NCDs).

AD remains so far, an incurable disease [1]. Most of its follow-up by the attending physicians is performed as an outpatient care in primary care facilities with occasional visits to specialised clinics. Its prognosis of clinical worsening may be predicted to a limited extent based on the disease onset. Nevertheless, our ability to affect its clinical course or slow down a neurodegenerative process leading to worsening of symptoms is exceptionally limited. This refers to changes of life style, innovative pharmaceuticals, psychosocial therapy modalities and physiatric methods. Dementia’s final outcome is a full-scale dependency of the patient from external help and supportive medical care in everyday living. It continues to affect a large share of elderly European citizens [2], creating many unmet medical needs. Many senior and retired citizens are living alone or in nursing homes. Their need for personalised medical care is far higher compared to an ordinary senior citizen suffering from one or few chronic NCDs but without dementia [3]. As AD progresses further, it leads to exposed dependency for care in everyday life routines, rapidly shrinking quality of life of the patient, and ultimately substantially decreased life expectancy.

BC itself dominates the landscape of female hormone-sensitive tumours. It has a vast burden of prevalence and incidence in the EU, although it has reached plateau levels in recent decades [4]. There were sharp advances in pharmaceutical innovation in the field of targeted oncology agents whose survival extension, depending on pathohistological aggressiveness and stage of malignancy at diagnosis, may range from several months up to a few years. Early diagnosis methods relying on advanced imaging diagnostics coupling mammography, computerised tomography (CT) and nuclear magnetic resonance (NMR) with artificial intelligence (AI) and liquid biopsy techniques are promising advances to make early and painless disease discovery a standard of care [5, 6]. Unlike many other solid tumours of inner organs, BC remains completely curable if discovered timely in the majority of the patients [7]. Its clinical impact on individual patients and their families and a social impact on the society lie primarily on the fact that it is mostly affecting working age women prior to menopause. Their anticipated life expectancy at the moment of diagnosis should have extended over decades [8]. Thus, costs due to absence from work (of employed women), their lost working ability and premature death are immense. For the survivors, other issues like reintegration to the job market and surgical correction of treatment effects via implant technologies remain an ongoing challenge.

PC dominates the morbidity patterns among male hormone-sensitive malignancies [9]. It is a frequent phenomenon among the senior males, mostly in their fifth, sixth, seventh and eight decades of life. Its clinical presentation is closely intertwined with benign prostatic hyperplasia, sharing some elements of clinical symptoms [10]. PC itself remains curable if detected in an early stage of disease evolution, encapsulated in the gland. Yet its clinical course is less favourable compared to BC, given the fact that it is not easily detectable and usually gives symptoms quite late. Such course leads to effectively lower survival rates, although there is an abundance of treatment options in the EU market. Its socioeconomic impact also refers to the fact that it partially affects male patients in maturity, much before their retirement age. This malignancy creates substantial shortening of survival and affects the working ability of an individual patient [11].

Targeted oncology agents and monoclonal antibodies can offer some moderate life extension gains, symptoms amelioration and quality of life improvement [12].

The important consequences of AD, BC and PC represent public health challenges that are topping the agendas of policy makers. Vast resources have been invested over the last two decades in these areas throughout the several consecutive EU science funding cycles [13]. Thus, retrospectively monitoring the outputs and impact of such investment in terms of innovation in pharmaceuticals, imaging and laboratory diagnostics, robotic medical care, rehabilitation and social reintegration of survivors is very important. Precise and carefully-measured output of such investment measured in terms of a variety of indicators might prove very meaningful for the European policy makers. It would enable understanding the added value of certain research streams and designated calls for funding within certain programs. It should also help authorities to make a reliable, evidence-grounded assessment of EU contribution to global innovation in these three biomedical areas. Consecutively, bottleneck inefficiencies and certain streams of funding gaining particularly low yield in terms of brand new knowledge generation, patents and scientometrics recognition worldwide could also be identified. This study aimed to analyse research funding streams in the fields of AD, BC and PC. The outcomes of this analysis could help shape current and forthcoming research programmes, re-allocating funding towards more productive and revenue-generating scientific [14] and industrial areas [15] if and when needed.

### Methodology

The JRC's Eu Reference Laboratory for alternatives to animal testing (EURL ECVAM) of the European Commission’s Joint Research Centre (JRC) in collaboration with EC Directorate General for Research and Innovation (RTD) initiated an orchestrated effort to define suitable indicators to assess the impact of EU-funded research in biomedical sciences, and assess the outputs and impacts of relevant research activities that benefited from EU financing. Reliable and measurable indicators should be suitable to retrospectively:

- monitor contribution of funded research to innovation
- monitor influence of funded research on public health trends, and
- inform future research and funding strategies.

As part of the activities, GOPA [16] was commissioned by the JRC to conduct a study on the impact of EU research investment in three distinct biomedical areas: AD, BC and PC. The primary outputs that were tracked, observed and measured throughout the development of a final list of 14 indicators, were real-world tangible outcomes of project spending. This exercise included patents, publications, clinical trials, innovative technologies, new diagnostic procedures, novel pharmaceuticals market launches, etc. Most of these outputs was largely achieved by means of Research and Innovation Actions (RIA) type of grants, and to a lesser extent European Research Council (ERC) grants, Marie Curie (currently "Marie Sklodowska-Curie Actions") grants, etc. Professional networking grants such as CSA (Coordination and Support Actions) and diverse mobility grants also played a vital role in European capacity building and mutual fertilisation of ideas, dissemination of skills and knowledge from centres of excellence in certain areas to the other institutions within their consortia. In order to decrease the misleading significance of the smaller mobility grants, in the context of this activity it was established to exclude any grants with an EC contribution below 200 000 EUR, regardless of their funding cycle and year of commencement and closure.

### Areas of biomedical research

The evolving landscape of European science and innovation funding was analysed with focus on AD, BC and BC. These were selected among an array of dementia entities and wide spectrum of oncology disorders, based on their perceived special significance in the European morbidity landscape. The goal was to reveal major visible and hidden patterns and trends over the past two decades.

AD was selected given the rising prevalence, incidence and overall burden of dementia among the European communities [17]. This strong epidemiological transition is largely driven by the profound underlying demographic change of the population ageing. Among several clinical types of dementia (AD, Vascular Dementia, Dementia with Lewy Bodies (DLB), Parkinson's Disease Dementia, Mixed Dementia, Frontotemporal Dementia (FTD), Huntington's Disease, Creutzfeldt-Jakob Disease), AD was the primary focus, given its epidemiological weight and clinical course, while other (non-AD) types of dementia were not covered.

Amongst the vast diversity of oncology disorders and solid malignant neoplasms, BC and PC were selected for their high prevalence, the variety of their pathohistological forms, both being hormone-sensitive tumours, and the fact that they dominate the oncology landscape among the ageing nations of Europe. BC tends to affect women of working age in their 30 s, 40 s and 50 s, leading to huge burden of absenteeism, opportunity costs of decreased working ability and premature mortality [18, 19]. The PC profile is similar, but to a slightly older cohort of adult men, mostly in their 50 s, 60 s or 70 s life decades [20]. Both diseases are largely curable when discovered at an early clinical stage, with rather low oncological grade and stage of disease [21, 22]. Given much easier and more efficient diagnostic screening and self-examination approach and advanced imaging diagnostics, female oncology survival rates in this entity tend to be far more favourable and promising. This is in contrast to male morbidity of comparable malignant tissue aggressiveness, given the fact that PC is mostly discovered accidentally and in a far later clinical stage of disease evolution [23, 24].

### Time coverage

The project covered (up to) 21 years of research, from the beginning of the FP5 funding programme in 1998 (official zero ground time of observation: 1st of January 1999) until the 31st of December 2019. The time coverage end date was set considering the financing mechanism and cycle, since the research projects that started in 2020 might not produce any or substantial results within 1-year frame. In addition, budgetary allocations for the fiscal year 2020 might have been legally adopted and officially released later, due to COVID19-caused administrative and legislative voting delays. However, this time reference (1998–2019) could not be covered in full for all the indicators constructed, due to data unavailability for some of these.

### Geographical scope and EU funding

The project focused on EU-funded biomedical research granted to beneficiaries from EU MS and other eligible consortia partners. The analysis also included the United Kingdom (UK), since it had been a member of the EU for the reference period, having participated in full financial capacity as a contributor and its science and research institutional network took part in the observed activities.

Biomedical research projects (for the three areas of research) funded through the EU research programmes FP5, FP6, FP7 and H2020 were covered.

### Research outputs

The project covered and explored all research outputs produced by the EU-funded projects in the domain of AD, BC, and PC, aiming to identify and track particularly the major outputs. The scope of the research outputs focused particularly on scientific and technological outputs (i.e. patents, diagnostic tools, approved drugs, treatments or medical devices, clinical trials) and dissemination outputs (i.e., publications and citations).

### Animal vs non-animal research methods and approaches or models

The analysis differentiated between projects (and their subsequent research outputs) that were exclusively based on animal approaches vs those based on non-animal approaches or a combination of these.

### Indicators

A set of 18 indicators was initially selected by the JRC at the start of the study, and their reliability was tested by GOPA in a methodologically robust manner. Indicators should be able to screen the past, present and future for an array of causal relationships and long-term trends in clinical, epidemiological and public health sphere, and ideally assess the broader socioeconomic impact of research on the society at large. These 18 indicators were stratified in six main categories as previously described [13]:

- funding and economic;
- scientific and technological;
- dissemination;
- regulatory and policy;
- public/social engagement; and
- education, training, and job opportunities.

A series of feasibility tests were conducted on the initially proposed 18 candidate indicators and allowed to finally came up with 14 of them. Ultimately, these 14 indicators, most being quantitative ones—while others were qualitative—proved their measurability, sensitivity and specificity to provide real-time prospective and retrospective tracking of EU-funded scientific outputs in the chosen biomedical areas.

Funding and Economic indicators represent a group of three, namely:

1. Number of projects in the selected biomedical research areas (AD, BC, PC) financed through FP5, FP6, FP7 and H2020;
2. Value of projects in the selected biomedical research areas (AD, BC, PC) financed through FP5, FP65, FP7 and H2020;
3. Value of co-financing from other institutions of projects in the selected biomedical research areas (AD, BC, PC) financed through FP5, FP6, FP7 and H2020.

The relevance of this group is its ability to provide precise structure in terms of the amount of spending across the three areas (AD, BC, PC) in four consecutive funding cycles FP5 FP6, FP7 and H2020, and participation of EU share in these grants’ fiscal flows.

The dissemination category consists of a total of two indicators, namely:

4. Number of publications from FP5, FP6, FP7 and H2020 projects in the selected biomedical research areas (AD, BC, PC); and
5. Number of citations of publications from FP5, FP6, FP7 and H2020 projects in the selected biomedical research areas (AD, BC, PC).

The first one gives insights about the scientific outputs from European research projects in the selected biomedical areas, while the latter shows the response by the targeted auditorium worldwide. Citation rates do not only reflect the wider readership of European science worldwide but also a decision to ground someone’s own further work on previously established EU evidence. This is the case since most established research groups tend to cite only short listed, carefully selected evidence, which was deemed to be particularly reliable and methodologically well-designed against a much larger body of screened publications [25]. In both areas, EU-funded science documented a strong growth with the citations increase being even more impressive. These are clear success stories in AD, BC and PC areas.

The scientific and Technological indicators group consists of a total of five members, namely:

6. Number of patents suitable to study the selected diseases and/or test new drugs, resulting from FP7 and H2020 projects, in the selected biomedical research areas (AD, BC, PC);
7. Number of projects targeting diagnostic tools established, resulting from FP5, FP6, FP7 and H2020 projects in the selected biomedical research areas (AD, BC, PC);
8. Number of projects targeting drugs, treatments or medical devices that have been developed/discovered, resulting from FP5, FP6, FP7 and H2020 projects in the selected biomedical research areas (AD, BC, PC);
9. Number of projects targeting clinical trials for new drugs initiated, resulting from FP5, FP6, FP7 and H2020 projects in the selected biomedical research areas (AD, BC, PC);
10. Number of projects targeting prevention measures, resulting from FP5, FP6, FP7 and H2020 projects in the selected biomedical research areas (AD, BC, PC).

Among these diverse and mutually complementary indicators, the number of patents is probably the most impactful, showing the structure and dynamics of essential innovation which has clearly accelerated and became more fruitful in the more recent funding cycles. In the context of this study, only patents derived from FP7 and H2020 were considered.

Regulatory and policy measures refer to the development of stringent national and EU strategies to combat the burden of AD, BC and PC through an array of accessible public insurance and health policy measures. These include the increase access and affrodability of medical care to ordinary citizens, by providing screening and preventive measures.

11. Contributions of EU-funded research in the three biomedical research areas (AD, BC, PC) to regulatory and policy actions (e.g. public health guidance values/opinions; regulatory policy actions; non-regulatory targeted policy actions).

These orchestrated efforts entailed adjustments of innovative good clinical practice (GCP) guidelines [26], which mostly refer to hospital setting and inpatient treatment.

Environmental, socioeconomic, early life conditions, individual actions, and medical care all interact to affect health. Regular national health interview surveys and the use of administrative data have provided data on morbidity, health services use, and some social determinants of health. One of the major uses of health indicators is to advocate on health issues. These measures shall be taken into account for decision-making targeting the improvement of the levels of health in the population and for reducing health inequalities. A second major use of indicators on public health is to achieve accountability. Such indicators represent a solid evidence base for governments, health professionals and agencies, and the general public, for information on risks, patterns, and trends related to health and whether or not expectations for performance are met.

Against the background presented for the three diseases covered by the study (see Introduction), it is clear that these represent a primary global concern, which requires local, national and international measures and policies (Table 1). Continuing to make progress in improving prevention, diagnosis, treatment and outcomes of these diseases, requires ultimately investing in an ecosystem of solid research and innovation, accompanied by efficient public policies that guarantee access to medical and care services.

**Table 1:** List of categories and indicators (14 in total) in the selected biomedical research areas (AD, BC, PC) financed through FP5, FP6, FP7 and H2020 ( between 1999 and 2020). As specified in the table, some indicators could not be analysed for the entire reference period/ EU-funded projects or all the selected biomedical areas.

Category	Indicators
Funding and economic	1. Number of projects
	2. Value of projects (Euros)
	3. Value of co-financing (Euros) from other institutions of projects
Dissemination	4. Number of publications
	5. Number of citations of publications
Scientific and technological	6. Number of patents suitable to study the selected diseases and/or test new drugs- only data from FP7 and H2020 projects were considered
	7. Number of projects targeting diagnostic tools established
	8. Number of projects targeting drugs, treatments or medical devices that have been developed/discovered
	9. Number of projects targeting clinical trials for new drugs initiated
	10. Number of projects targeting prevention measures
Regulatory and policy measures	11. Contributions of EU-funded research to regulatory and policy actions (e.g. public health guidance values/opinions; regulatory policy actions; non-regulatory targeted policy actions)
Main public health trends	12. European indicator: Public health trends (i.e., deaths per 100 000 inhabitants and incidence per 100 000 inhabitants) between 2011 and 2019
Education, training, and job opportunities	13. Number of staff/researchers employed—only data from H2020 were considered
	14. New learning opportunities

Tracing the main public health trends for the three biomedical research areas is, therefore, an important contextual indicator the research outcomes can be referenced against and, as well, a tool that can support the planning of research funding. In our study, Major Public Health Trends is addressed as a category and indicator on its own:

12. European indicator: Public health trends (i.e., deaths per 100 000 inhabitants and incidence per 100 000 inhabitants) on selected diseases (i.e., AD, BC, PC) between 2011 and 2019.

For this indicator, only this eight-year time frame was considered as…

Education, Training, and Job Opportunities indicators refer to two indicators:

13. Number of staff/researchers employed in the biomedical research projects on AD, BC, PC in H2020; and
14. New learning opportunities resulting from FP5, FP6, FP7 and H2020 projects in the selected biomedical research areas (AD, BC, PC).

The EU policy agenda has put a strong emphasis on networking grants, in order to increase mutual fertilisation of ideas and capacity building across Europe [27]. These three biomedical areas had an array of calls and topic programmes among which the most notable were: Marie-Curie Action: ‘Intra-European Fellowships for career development’ (FP7); Marie-Curie Action: ‘Initial Training Networks’ (FP7); Marie Sklodowska-Curie Action: ‘Innovative Training Networks’ (H2020) and Strategy for exploitation of research results funded under Euratom Research and training Programmes in the field of radiation protection. With regards to employment dynamics and job openings in these scientific areas, official data records are far less clear, and incomplete and for this reason no indicators about job opportunities were considered in this study.

## Results

### Funding and economic indicators

Over the two decades studied, the EC has been steadily increasing both the volume of projects being funded and the amounts of financial resources being allocated.

### Number of projects

The entire quantity of all grants being retained for funding in these three biomedical areas on the 1999–2019 timeline, were 235 in AD, 283 in BC and 72 in PC. The count of projects being financed has been growing steadily over the years. For AD it meant almost five times more grants, and in BC three times more grants in FP7 in comparison to FP5. H2020 brought a minor contraction in AD, while BC and PC consolidated at approximately similar levels. (Please see Table 2.)

**Table 2:** Number of projects funded across all four cycles and the associated fiscal flows (indicator 1 and 2)

	FP5	FP6	FP7	H2020
Number of projects				
AD	22	37	107	69
BC	27	57	93	106
PC	9	14	26	23
Total cost (million EUR)				
AD	44.35	218.96	451.87	272.72
BC	54.31	247.22	334.97	345.40
PC	15.14	52.51	68.65	77.40
EC contribution (million EUR)				
AD	31.19	164.17	301.48	220.26
BC	38.16	181.81	255.58	329.36
PC	11.83	42.41	62.77	61.82

Source: authors’ elaboration based on data processing results

Additionally, in this analysis we considered what specific methodological approaches were used in these projects, to distinguish those based exclusively on the use of animal models from those that accounted for a combination of different methodological approaches (e.g., in vitro, in vivo and/or in silico/computational methods or models).

A share of 23% out of the total number of projects under the AD biomedical research area made exclusive use of an animal model. For AD the share is the highest among the three biomedical research areas, followed by 15% for BC and 13% for PC, among the projects that relied only on methodological approaches using animal or animal-derived materials. The share is consistent, and the order is the same, in the case of the number of projects by biomedical research area which followed approaches including animal or animal-derived models in combination with other models (45% for AD, 39% for BC and 33% for PC). Add fig?

### Value of projects

From a financial point of view, this growth was exponential, rising ten times more in AD from EUR 44 million in FP5 to EUR 452 million in FP7. Again, given the significant lack of tangible output in this area in terms of causal treatment innovation, priorities on EC’s Agenda were adapted in H2020, resulting in a reduced amount of spending on AD (EUR 272 million). In BC and PC areas, the expenditure dynamic was an upward one, without reductions in fiscal flows. BC funding rose from EUR 54 million in FP5 to EUR 345 in H2020 and PC from EUR 15 to EUR 77 million.

Finally, yet importantly, we need to emphasize the entire volume of funding which has reached approximately EUR 988 million in AD (EC contribution of EUR 717 million), EUR 979 million in BC (EC contribution of EUR 805 million) and EUR 214 million in PC (EC contribution of EUR 179 million). The remaining amounts were co-financed by a variety of beneficiaries ranging from the large industry, universities’ own endowment and a variety of national or multilateral funding agencies.

### Value of projects’ co-financing

The financial shares (i.e. total volume and share of EC contribution) for the projects using exclusively animal models are fairly consistent with the respective shares in terms of number of projects in total. Namely, 13% of the total volume of PC projects belonged to projects that followed exclusively a methodological approach using animals or animal-derived materials, and respectively 13% for BC and 27% for AD (their share as per number of projects out of total being 13% for PC, 15% for BC and 23% for AD).

As nominal values, in total, the volume of projects that made use exclusively of animal models was approximately EUR 408 million. In other words, about 19% of the total volume of all projects developed research outputs based on animal or animal-derived materials. However, there is a noticeable gap between the nominal values allocated to the projects using exclusively animal models among the three biomedical research areas. Most noticeable is the difference in nominal values between the AD and BC areas, which although summed similar total volumes (EUR 988 vs EUR 979 million, respectively), the volume corresponding to projects using exclusively animal models was approximately EUR 115 million less for BC projects than for AD projects. (Please see Table 3.)

**Table 3:**
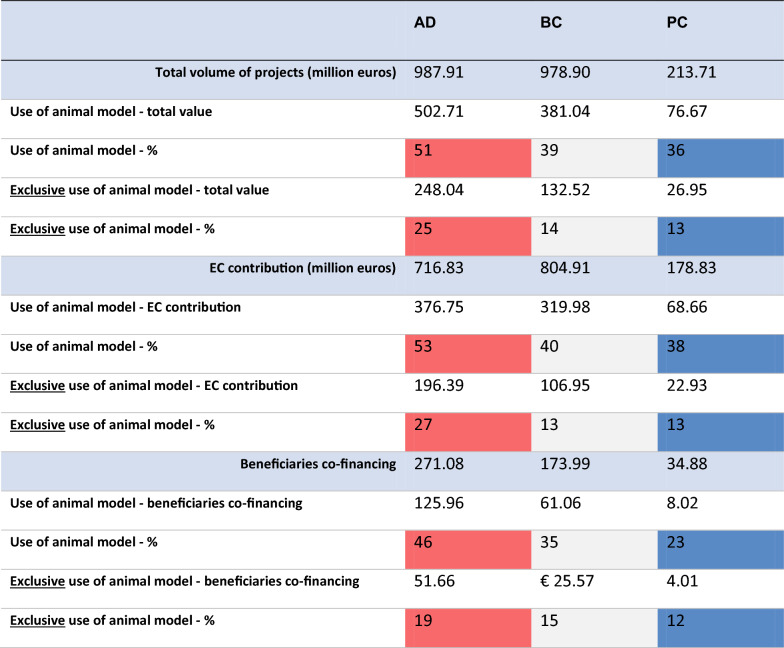
Total volume, EC contribution and value of co-financing from other institutions (indicators 2 and 3)

Source: authors’ elaboration based on data processing results

### Dissemination indicators

#### Publications

An overall output of the funded grants in scientific terms, of quantity and quality of published evidence is shown in Table 4. The highest number of publications corresponds to AD-related projects (6,584 publications), followed closely by BC (6,116 publications). An important difference between the number of publications produced from AD- and BC-related projects compared to the PC projects is noticeable (only 1 372 publications). The lower number of publications resulting from PC-related projects can be attributed partly to the lower number of projects in this biomedical research area (only 12% of the total number of projects analysed). However, the average number of publications per project by biomedical research area is also lower for the PC projects (19.1 for PC vs 21.6 for BC vs 28 for AD).

**Table 4:** Number of publications by their type (indicator 4)

	No of publications	Article materials	Conference materials	Editorial materials	Book materials
AD	6.584	5.990	262	197	135
BC	6.116	5.412	470	134	100
PC	1.372	1.189	129	35	19
Total	14.072	12.591	861	366	254

Source: authors’ elaboration based on data processing results

It can be observed that the publishing output was heavily dominated by full-length peer reviewed articles. The bibliographic structure of created evidence consisted of original research and review pieces in a share of 65–85%, depending on the biomedical research area. This was followed—a far lesser extent—by books, monographies, theses, and conference and editorial materials.

Another important trend is the rise of publications output through the consecutive funding cycles, which by far exceeds both the amounts of projects being funded and fiscal flows allocated to their realisation. (Please see Table 4.)

This rise is particularly noticeable for FP7 to H2020 programmes, for which the input data used for the calculation of this indicator was also of higher quality, compared with the publications’ data for FP5 and FP6, particularly in terms of comprehensiveness and comparability. This is due to the fact that at the time when FP5 and FP6 programmes were launched (during the’90 s and early’00 s) worldwide web capacities where by far underdeveloped, in comparison to modern day perception. Electronic -based publishing models were only pioneered as early models, while hard-copy, paper-based publishing of scientific articles and study monographs were mostly prevailing both in Europe and globally. In later years many of FP5- and FP6-attributable publications ended up archived in traditional libraries. A certain share of such evidence was scanned and made public, but only as a photographic textual content that is accessible for human reading today, nevertheless not to extracting by AI algorithms and indexing databases in their full-text content. This means that FP5 and FP6 low productivity impressions are misleading due to the substantially limited abilities to search through this body of evidence via contemporary librarian software tools.

The ascending trend is interrupted in 2013–14, when a slight decrease in the number of publications is registered. This can be attributable to the gap between the two financing programmes; i.e. FP7 and H2020. In addition, a decreasing trend is observed from 2019 and, more drastically, starting with 2020. The decrease can be attributable to the time lag between the projects’ outcome and publications, but also to an increased focus on COVID19-related research in 2020 and 2021 or to a change in the goals of the projects.

#### Citations

**Fig. 1:**
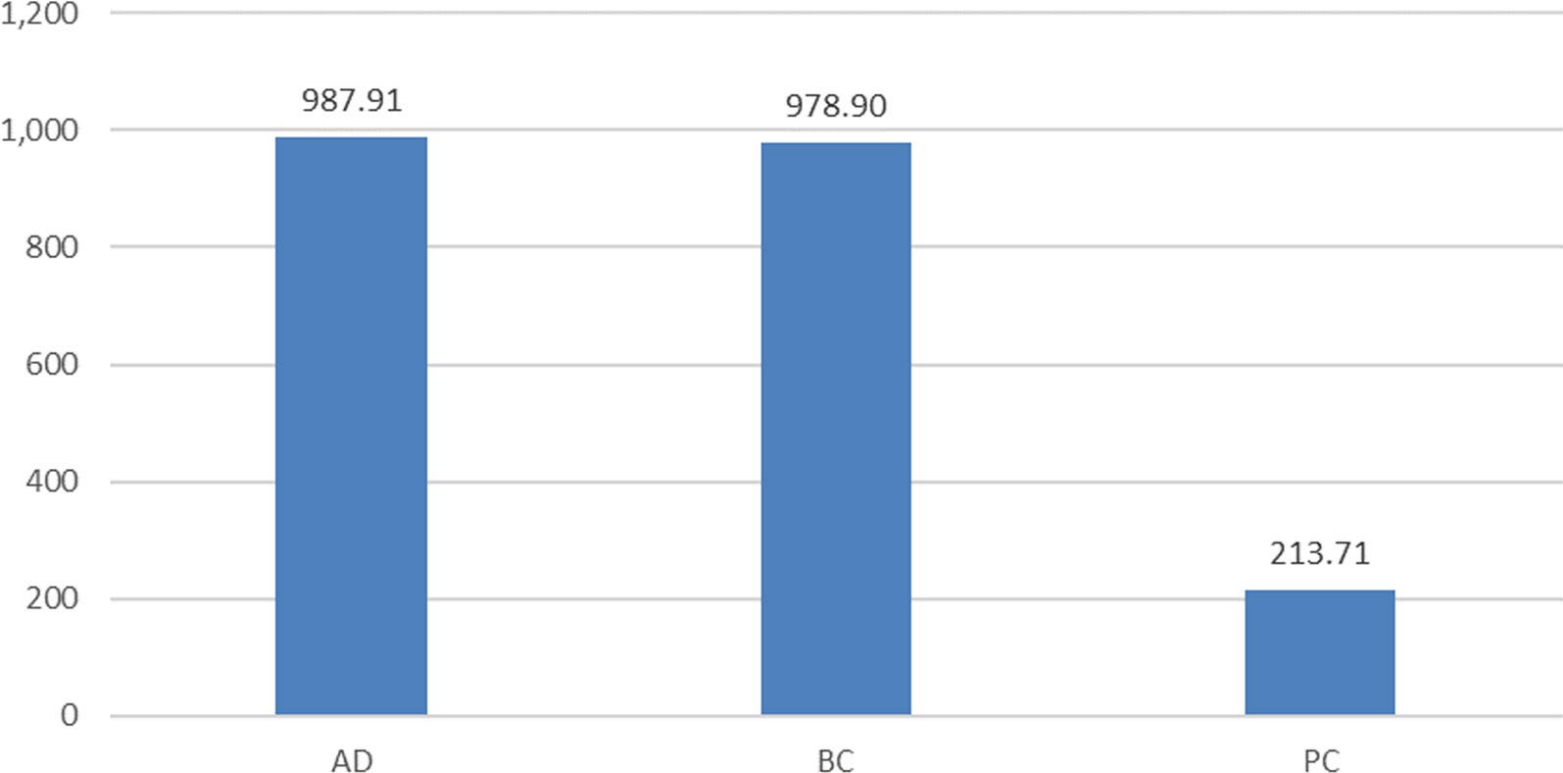
Total volume of projects by biomedical research area—million EUR—indicator 1

With regards to citations, it can be stated that the impact of publications resulting from the projects analysed had a high impact, registering a high number of citations. However, it should be noted that the analysis does not differentiate between self-citations and third parties’ citations, and the figures resorted here include all citations, based on the identified source, i.e. Altmetrics. On average, 41.31 citations per publication can be derived, with the highest impact registered by BC-related publications with 44.39 citations per publication. If the average citations per publications are calculated only for ‘article materials’ the impact registered is even higher (reaching 48.44 citations per article for BC). Similar trends, as for the number of publications by year, can be observed for the number of citations. (Please see Fig. 1.)

**Fig. 2:**
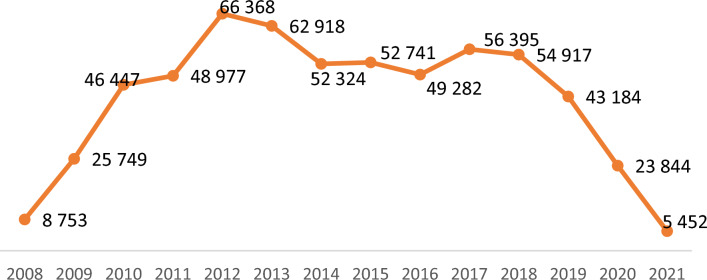
Number of citations of publications, 2008–2021 (indicator 5). Source: authors’ elaboration based on data processing results

These citation-based indicators clearly point out to the rising global competitiveness of European science in these three biomedical areas. Their ability to attract targeted audience worldwide, both expert and, to a lesser extent lay audience, was exceptionally successful. At the same time, it continues to outreach the quantities of funding allocation, since it exceeds by far the parallel growth in cumulative number of projects and the amounts being at the disposal of respective consortia, which delivered these results. (Please see Fig. 2.)

### Scientific and technological indicators

#### Patents

A total of 179 patent applications were filled from projects financed through FP7 and H2020 grants with an EC contribution above EUR 200 000. The highest number of patent applications correspond to the BC biomedical research area (92 patent applications), followed by AD (68) and PC (19). As percentage share, 38% of the patent applications for AD research area (i.e. 26 out of the 68) correspond to projects that made an exclusive use of animal research models. The percentage share of patent applications resulting from projects that made an exclusive use of animal research models is considerably lower for the BC and PC biomedical research areas, which represented 24% (22 patent applications) and 5% (1 patent application) respectively. (Please see Table 5.)

**Table 5:** Number of publications throughout the research cycles (indicator 4)

	FP5	FP6	FP7	H2020
AD	47	260	3.878	2.399
BC	12	366	3.725	2.013
PC	1	143	635	593

Source: authors’ elaboration based on data processing results

#### Diagnostic tools

Out of the total 102 projects targeting the development of diagnostic tools, more than half (57 projects) correspond to the BC biomedical research area, followed by AD (28 projects) and PC (17 projects). Only four projects followed a methodological approach using exclusively an animal model (3 projects for BC and one project for AD) out of the 16 projects that made use of animal models overall. The great majority of the projects (86 projects) made use of other methodological approaches, with in-silico/AI models used exclusively, surpassing the ones that used human models exclusively (35 vs 33 projects, respectively). (Please see Table 6.)

**Table 6:** Number of patents (indicator 6)

	No of patents	Animal models	Animal models (exclusive)	Other (non-animal) models	Human model (exclusive)	In-silico/AI model (exclusive)
AD	68	33	26	35	15	4
BC	92	35	22	57	22	27
PC	19	6	1	13	9	2
Total	179	74	49	105	46	33

Source: authors’ elaboration based on data processing results

Three categories of diagnostic tests were observed in the analysed array of EU-funded grants in the biomedical areas of AD, BC and PC. First are imaging diagnostics ranging from traditional methods like Roentgen’s X-rays-based mammography, PET (Positron emission tomography) scans and NMR [28]. A variety of innovative grants brought a movement of the diagnostic frontier forward than ever before in clinical practice. They have managed to increase both the sensitivity (ability to accurately recognise and measure true positive rate) and specificity (ability to prove true negative rate). These gains were achieved with a variety of creative approaches, such as radioactivity-labelled substances such as monosaccharides used in PET scan to measure blood perfusion and brain activity via PET scan. In H2020 years, early AI projects tested machine learning ability and deep algorithms. This approach was based on the fact that AI was thought to be trained on dozens of thousands and hundreds of thousands actual patient images, representing diagnostically confirmed tumours and healthy organs. In a later stage, AI exposed some degree of capability to distinguish breast malignancies and nodal changes at such an early stage that would be hard to catch by human eye observation of an experienced radiologist or oncologist [29]. The vast majority of these innovative imaging diagnostics solutions, frequently involving nanotechnology and chip devices, were explored and developed in human patients. For these purposes, animal models are less convenient due to substantial anatomical differences.

The second group of diagnostic tests are laboratory tests. In case of AD, these are mostly based on specimens of cerebrospinal liquid and in oncological indications on various sorts of blood samples. Among truly innovative solutions, a grant proposing AD diagnostics based on a marker detected in a singular blood test should be highlighted [30]. A variety of approaches were proposed to develop new tumour markers and novel markers of neurodegenerative processes, leading to amyloid formations appearance in the brain and consecutive dementia. Some of these tests, despite rare exemptions, were tested on laboratory animals presenting disease models, such as rodents suffering from transplanted BC. Given the fact that rat- and mouse-based dementia models can be created, these modelling frameworks were used as well.

The third and least applicable set of diagnostic tools are actually psychometric questionnaires. Coupled with anamnestic (history of disease) data and standard neurological examination by the attending physician, these standardised and validated surveys remain the mainstay of dementia diagnostics even today. There were few funded attempts targeted to make psychological and neurological diagnostic tools more efficient, since these techniques belong to traditional clinical medicine and experienced rather little essential change during the 90’s, 00’s and 10’s. Minor improvements were indeed noted in the field of quality of life and symptom scales in terms of validation and standardisation of these questions and answers in various world languages and their adaptation to particular diseases [31].

In our observation of the three selected biomedical areas, a total of 102 grants explored novel diagnostic approaches, out of which six ended up with truly successful commercialisation and marketing approval of such medical devices or advanced laboratory tests [32].

#### Drug discovery

Across FP5, FP6, FP7 and H2020, 64 projects targeted the development, testing or repurposing of drugs, with the great majority of these targeting drug development. (Please see Table 7.)

**Table 7:** Number of projects that targeted the development of diagnostic tools (indicator 7)

	No of projects targeting diagnosis	Animal models	Animal models (exclusive)	Other (non-animal) models	Human model (exclusive)	In-silico/AI model (exclusive)
AD	28	5	1	23	10	8
BC	57	7	3	50	18	22
PC	17	4	0	13	5	5
Total	102	16	4	86	33	35

Source: authors’ elaboration based on data processing results

The typical drug development life cycle begins with the preclinical drug testing on microbes, human cell cultures and animal models. If there is promising evidence in support of therapeutic efficacy and acceptable safety/toxicity profile, it is continued through clinical development in four phases (I–IV). The biomedical fields of AD, BC and PC have indeed brought upon a significant number of new drug approvals for marketing in Europe during the period 1999–2019. It is important to note that it is relatively difficult to establish a causal connection among certain EU-funded grants and supposedly consecutive new drug launch. This is the case for several reasons. Firstly, the fact that essential pharmaceutical innovations assume synthesis and development of the brand new active ingredient. Such an active compound in certain illness should preferably expose a novel molecular mechanism of action in terms of its pharmacodynamic profile. This sort of innovation remains exceptionally expensive and requires a lengthy time investment. Traditionally, only one out of 10 000 drug candidates manage to survive the race from the potential chemical candidate to the market launch following successful collection of Phase III trial evidence [33]. Furthermore, single grant budget within the H2020 cycle ranged on average around few million EUR [34]. Establishment and launch of the brand new representative of new pharmaceutical Anatomical Therapeutic Chemical (ATC) classification system used to cost at least few hundred times more [35]. Thus, the potential for deep innovation, particularly in essentially incurable diseases such as AD, BC, PC assumes private–public partnership. This criterion was fulfilled by many project consortia involving a large number of universities and research institutes across Europe, plus a big pharma company. Such grants tended to exceed substantially an average Research and Innovation Actions (RIA) grant value and ranged between EUR 15–50 million. In addition, it was noted that such large, well-funded consortia tended to generate significant output in terms of new knowledge frontiers, breakthrough technologies and, ultimately, some effective therapeutic agents.

This blossoming fertility of innovations was mostly noted in nanotechnology-related approaches, monoclonal antibodies and targeted oncology small molecular entities indicated in BC and PC. Success rates in treatment of AD or even achievement to postpone its symptoms worsening or extended survival were exponentially less successful. The traditional landscape of treatment options for AD consists of currently EC-listed cholinesterase inhibitors (ChEIs) for patients with mild to moderate disease (with the exception of donepezil, which is also approved for moderate to severe disease), and the N-methyl-D-aspartate (NMDA) receptor partial antagonist memantine approved for use in moderate to severe AD [36]. So far, these options offer temporary symptomatic relief to patients, rather than anything approaching effective amelioration of AD pathology. Immunotherapies targeting amyloid beta designed to enhance and facilitate amyloid beta (AB) clearance from the brain are undergoing development. Yet, there have also been significant high-profile failures of drugs in late stage clinical trials that could potentially alter the future landscape of novel treatment through their inefficacy [37]. Examples of few such tested agents are: flurbiprofen (tarenflurbil), tramiprosate, latrepirdine, semagacestat, IVIg, Cerebrolysin, EGb 761, docosahexaenoic acid (DHA).

Globally leading regulatory agencies and core members of the International Council on Harmonisation of Technical Requirements for Registration of Pharmaceuticals for Human Use (ICH), the US based FDA (Food and Drug Administration), the European EMA and the Japanese PMDA-MHLW have jointly established a set of guidelines for the clinical evaluation of drugs intended for the treatment of AD [38].

Aligned with these ICH adopted FDA-EMA-PMDA Guideline initiatives, there was a large set of industry-led submissions for marketing and/or reimbursement approvals to these and smaller national medicines agencies across EU, during the observed two decades. There were many historical withdrawals of pharmaceutical agents not fulfilling evidence-based medicine criteria for efficacy, and many rejections by regulatory bodies. There are several dozens of potential therapies for AD in the pipeline of R&D investment by the global pharmaceutical manufacturing industry, ranging from Phase I to Phase IV. Yet, so far, there remain currently only six medicines approved in the European Union and the USA with supposedly curative treatment role indicated for AD, or at least able to slow down progression and worsening of the symptoms, namely: Donepezil Aricept^™^, Donepezil hydrochloride, Eranz^®^, E 2020 (donepezil hydrochloride) Galantamine Razadyne^™^, Reminyl^™^, Nivalin^®^ (galantamine) Memantine Ebixa^™^, Namenda^™^, Axura^®^, Akatinol^®^, Memary^®^ (memantine hydrochloride) Rivastigmine Exelon^™^, Rivastigmine tartrate, Rivastach^®^ Patch, Prometax^®^, SDZ ENA 713 (rivastigmine tartrate) Suvorexant Belsomra, MK-4305 (suvorexant) Tacrine Cognex^™^ (tacrine).

The field of oncology therapeutics in the European countries, and the attending clinicians’ prescribing practice and pharmaceuticals dispensing, are mostly regulated via the European Society for Medical Oncology (ESMO) guidelines and particular BC guidelines devoted to itself in its many pathohistological forms.

Furthermore, there are various national guidelines, which are more or less aligned with these general framework documents. They mostly refer to treatment algorithms, where certain types of tumours, such as breast adenocarcinoma, are graded for biological aggressiveness, based on a tissue sample taken either through needle biopsy or via full scale tumour excision during the surgical intervention. Alongside with imaging diagnostic techniques and tumour markers laboratory tests (explored elsewhere in this document), breast tumour is staged depending on the scale of disease spreading, ranging from local, regional lymph nodes, up to distant systemic metastasis.

It is important to understand that the vast majority of pharmacological therapeutic agents named cytostatic or cytotoxic compounds were discovered and approved at most of the European national markets long before 1999. Most of the classical cytostatic—chemotherapeutic agents to treat malignant neoplasms such as platinum salts (cisplatin), taxanes, lomustine, carmustine and many others with diverse modes of action, were invented and released to clinical practice between the 60 s and the 80 s.

Therefore, our search on BC-related pharmaceutical innovation focussed literary on essential innovation—drugs with innovative mode of action, safety or efficacy to some extent unseen or difficult to compare with their historical counterparts and predecessors.

This refers mostly to small molecules such as targeted oncology drugs, monoclonal antibodies, biological, biosimilars or occasionally some auxiliary agents, which might substantially improve accuracy (sensitivity/specificity) of diagnostic tests (such as radioactive contrast solutions mentioned above) and/or decrease toxicity / adverse events (antiemetics such as ondansetron, increasing the duration of treatment cycles due to improved tolerance).

In particular, targeted oncology agents are those that exploit the benefits of genotyping procedures, HLA markers identification and PCR analyses used to identify biomolecular targets for medicines on the surface of cancer cells or within their biochemical pathways.

The below list of approved medicines in Europe serves as a convenient cross-section of typical, most recognised and prominent representatives of essential innovation over the time period analysed and for the area of BC. Moreover, this list is not exhaustive since the search, by using the following limitations on official EMA website ‘Human AND European public assessment reports (EPAR) AND Authorised AND 1st January 1999-31st December 2019’, produces a total of 498 results. Yet, these are not 498 different chemical compounds, but rather 498 different brand name drugs with frequently the very same active ingredient within different pharmaceutical preparations.

Many of these pharmaceutical preparations are actually new brand names of old patented drugs, which are placed in the market as generic or copycat products after the patent expiry of the original agent. Nineteen truly innovative drugs have been marketed in Europe over the observed period, indicated to treat BC, namely: Tukysa^®^—tucatinib Kadcyla^®^—T-DM1 (ado-trastuzumab emtansine) Herceptin^®^—trastuzumab Ibrance^®^—palbociclib Perjeta^®^—pertuzumab Trodelvy^®^—Sacituzumab govitecan Enhertu^®^—trastuzumab deruxtecan Tecentriq^®^—atezolizumab Keytruda^®^—pembrolizumab Perjeta^®^—pertuzumab Fareston^®^—toremifene Kisqali^®^—Ribociclib Nerlynx^®^—neratinib Tyverb^®^—lapatinib Faslodex^®^—fulvestrant Docetaxel Zentiva^®^—docetaxel (previously Docetaxel Winthrop) Avastin^®^- bevacizumab;

There are substantial similarities in clinical pharmacology approaches to treatment protocols in BC and PC with few other major solid tumours. There was a substantial wave of essential innovation, mostly led by pharmaceutical industry and medical device manufacturing industry—over the past twenty years. The ESMO guidelines and, in particular, guidelines devoted to PC are presenting the milestones of pharmacological development.

Similarly, as with other solid tumours, the therapeutic approach will largely depend on the stage and grade of the tumour itself at the moment of diagnosis and, therefore, its prognostic scores.

In line with this, the following modes of action will be primarily considered: small molecules belonging to the targeted oncology drugs, monoclonal antibodies, biological, biosimilars, etc. Just like in the previous medical area (i.e. BC), particular targeted oncology agents are those that exploit the benefits of genotyping procedures, HLA markers identification and PCR, used to identify biomolecular targets for medicines on the surface of cancer cells or within their inner biochemical pathways.

As for BC, the non-exhaustive list below provides a convenient cross-section of typical, most recognised and prominent representatives of essential innovation, given the time span involved and area of prostate malignant neoplasms. By using the following limitations on the official EMA website ‘Human AND European public assessment reports (EPAR) AND Authorised AND Marketing Authorization Date: between 1st January 1999 and 31st December 2019 AND Prostate/Prostatic Cancer’—produces a total of 728 results, considering that these are 728 different brand name drugs with frequently the very same active ingredient within different pharmaceutical preparations, and many of them are actually new brand names of old patented drugs which are placed to the market as generic or copycat products after the patent expiry of the original agent.

Thirteen truly innovative drugs or technologies have been granted marketing authorisaton in Europe, over the observed period, indicated to treat PC namely: Zytiga^®^—abiraterone Lynparza^®^—olaparib Xtandi^®^—enzalutamide Firmagon^®^—degarelix Tookad^®^—padeliporfin Provenge^®^—sipuleucel-T (PC Vaccine [39]) Erleada^®^—apalutamide Nubeqa^®^—darolutamide.

#### Clinical trials

Chemical substances with the potential to assist prevention, screening, diagnostics, treatment or rehabilitation of human diseases and disorders undergo complex and lengthy biomedical and clinical research. Early stages (pre-clinical development) involve biomolecular testing, testing on microbial prokaryotic cells such as bacteria; testing on healthy/normal and sick/disease eukaryotic animal cells, tissues, organs, or entire living organisms known as animal models (mice, rats, rabbits, dogs, cats, monkeys, etc.). If the tested substance survives the comprehensive testing on efficiency to control targeted disorder (such as biological membrane electric potential, blood sugar levels, pain perception, malignant cells survival, etc.) and safety (acute and chronic toxicity, mutagenicity, teratogenic potential in pregnancy, oncogenesis, etc.), it can then enter the clinical stage of development.

With regards to projects that initiated clinical trials, grants funded in BC with partial of full participation from the EC grew from 9 to 27. PC stagnated from three to only two, while AD demonstrated a temporary peak rising from 7 in FP5 to 13 in FP7 and returning back to same plateau of 7 trials in H2020. (Please see Table 8).

**Table 8:** Number of projects that targeted drug development, testing or repurposing (indicator 8)

	No of projects targeting drug development, testing or repurposing	Animal models	Animal models (exclusive)	Other (non-animal) models	Human model (exclusive)	In-silico/AI model (exclusive)
AD	23	13	5	10	7	1
BC	30	23	14	7	4	0
PC	11	5	2	6	4	1
Total	64	41	21	23	15	2

Source: authors’ elaboration based on data processing results

Phase 0 and Phase I clinical trials are being conducted in healthy volunteers, mostly to test pharmacokinetics and pharmacodynamics of a given drug and plausible toxicities / adverse events.

Phase II clinical trials are tested in a rather small sample size of patients suffering from targeted disease (dementia, diabetes, etc.). These sample sizes usually range from several dozen up to 300 patients in diseases with massive pool of prevalence and incidence such as NCDs.

Phase III clinical trials are the pillar of clinical development and the last stage prior to submission of medical documentation-knowledge acquired on a given drug for the assessment by regulatory bodies aimed at marketing approval. This third stage of clinical trials assumes large sample sizes, unless the targeted disease is exceptionally rare (“orphan drug” designations) and may count up to several thousands (3 000 for example) of patients. Since so large sample sizes are difficult to recruit from a single tertiary care university hospital or region, they are mostly recruited globally through an array of countries and nations worldwide (“global trials”).

Randomized Controlled Clinical Trial (RCT) remains the mainstay of this methodology and the most reliable framework for acquiring reliable knowledge. Thus, most of these Phase II and Phase III trials are as per their scientific study designs RCTs of various forms and sizes and ambitions.

This implies the presence of an experimental patient group (receiving the *new agent*—plausible curative medicine) and a control group (receiving placebo) and double blinding of the study investigators (attending clinical physicians in most cases) and the patients, so no party is aware of who is receiving the active drug and who is receiving the placebo.

Other study designs may be applicable in an array of peculiar scenarios with rare diseases or rare indications within the single disease areas (such as subgroups of patients with specific genetic mutations allowing them to be successfully treated) or in testing of array of brand new, expensive drug launches, such as small molecule targeted oncology (typical example: Imatinib) or monoclonal antibodies or Advanced therapy medicinal products (ATMPs).

**Fig. 3:**
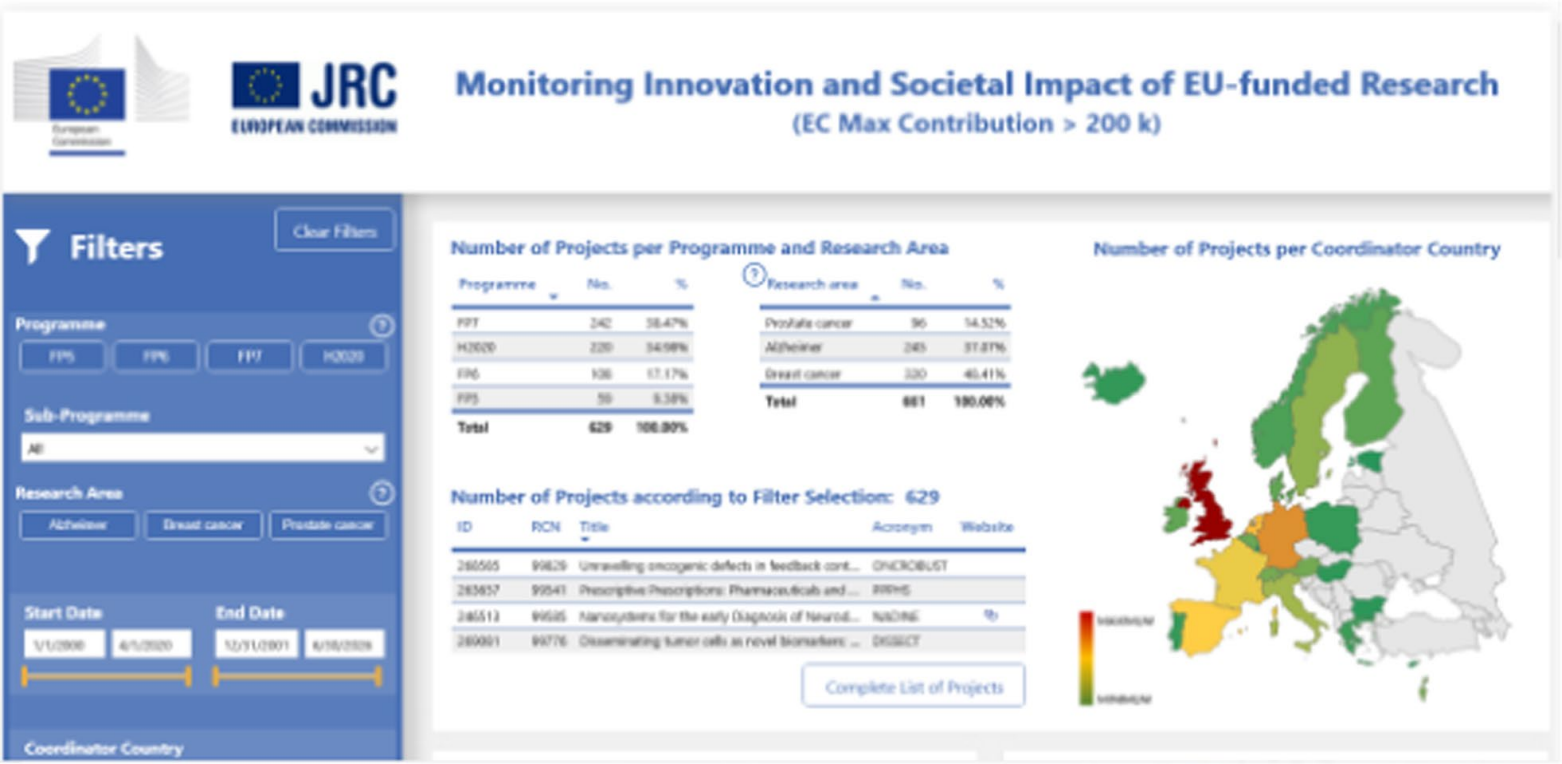
Interactive Software Platform created to track AD, PC and BC trends in real time at EU level

Phase IV clinical trials are so called post-marketing explorations. They take place in a much more massive sample size, once the drug has already begun to be prescribed and dispensed to the patients (in many countries partially or fully reimbursed from public or private health insurance funds). This means that many unknown or rare potentially life-threatening toxic effects—drug-attributable adverse events, shall be recognised and identified only when dozens and hundreds of thousands of patients receive a given drug. It may take several years to firmly approve and acquire evidence for a causal connection (such as between angioedema of neck tissues and angiotensin inhibitors / angiotensin receptor blockers, gold standard therapies in hypertension). (Please see Fig. 3.)

### Regulatory and policy measures

Projects analysed in this study were also screened, based on expert considerations, for the inclusion of preventive measures -considering whether they are primary, secondary or tertiary type of prevention.

A total of 45 projects targeted prevention measures across the three diseases over the period 1999 -2019, with the highest number (26) in BC.

A clear trend along the different research programmes cannot be identified: for FP7 the number of projects targeting prevention measures was similar to FP5, though comparing FP6 with FP5 an increase was noted. Overall, H2020 concentrates the largest number of projects targeting prevention measures from all research programmes. (Please see Tables 9 and 10).

**Table 9:** Number of projects that had initiated clinical trials (indicator 9)

	FP5	FP6	FP7	H2020	Total
AD	7	7	13	7	34
BC	9	16	16	27	68
PC	3	6	6	2	17

Source: authors’ elaboration based on data processing results

**Table 10:** Number of projects targeting prevention measures (indicator 10)

	No of projects targeting prevention measures	Animal models	Animal models (exclusive)	Other (non-animal) models	Human model (exclusive)	In-silico/AI model (exclusive)
AD	14	3	2	11	5	5
BC	26	7	3	19	9	4
PC	5	0	0	5	2	0
Total	45	10	5	35	16	9

Source: authors’ elaboration based on data processing results

Overall, a total of 29 grants account for the most successful preventive measures in terms of their cutting-edge innovation outputs (see Table 11).

**Table 11:** Number of projects targeting prevention measures throughout the research programmes (indicator 10)

	FP5	FP6	FP7	H2020	Total
AD	2	1	4	7	14
BC	4	9	3	10	26
PC	1	3	1	0	5

Source: authors’ elaboration based on data processing results

### Major public health trends in AD, BC and PC across the EU

The major public health trends in the EU analysed over the period 2011–2019, are characterised by a steep increase in the number of deaths per 100 000 inhabitants (43%), attributable to AD. Overall, from the three disease areas covered by the project, AD is the disease with the highest increases observed for the different indicators measuring public trends. Along the deaths per 100 000 inhabitants, the incidence per 100 000 inhabitants has also increased by 16%.

Deaths per 100 000 inhabitants caused by BC and PC increased at a slower pace over 2011–2019, by 3% and 6%, respectively. However, for BC, the increase in the number of deaths was higher than the increase of the incidence per 100 000 inhabitants (only 1%). Nevertheless, for PC, the incidence per 100 000 inhabitants increased by 12% over 2011–2019. (See Tables 12 and 13).

**Table 12:** Most successful grants in terms of their cutting-edge innovation outputs, commercialisation through patents and tangible market penetration of some of the discoveries

	Project acronym (and ID)	Diagnostic tool	Model	Total cost (million EUR)	EC contribution (million EUR)	Application
AD	ADDIA (674474)	Peripheral blood diagnostic biomarker kit	h	4.99	4.99	Diagnostic tools
AD	VERDAD (223671)	World’s first in-vitro diagnostic (IVD) blood test for accurate AD diagnosis—PreADx	h	3.37	2.36	Diagnostic tools
AD	NeuroLF (229999)	Small head adjusted PET scanner development	h	3.20	2.25	Diagnostic tools
BC	MAMMA (628919)	Spatio-temporal modelling MRI mediated substantial improvement in diagnostic accuracy and efficiency	AI	0.24	0.24	Diagnostic tools
BC	MammaPrint (672570)	Evolutionary BC Diagnostic Test—(MammaPrint^®^), as described in WO2002/103320	h	4.05	4.05	Diagnostic tools
PC	NANOZ-ONIC (682286)	Development of an innovative biosensor for the non-invasive, painless and real-time detection of volatile biomarkers in the exhaled breath of patients	A, AI	1.92	1.92	Diagnostic tools
BC	EPITRON (518417)	This discovery has later led to the establishment of truly innovative targeted oncology TNF drugs	a	13.70	10.90	Drug development or testing
BC	TRIDENT (37686)	This discovery has later led to the establishment of truly innovative targeted oncology TNF drugs	a	2.50	2.07	Drug development or testing
BC	MET-CANCER THERAPY (509804)	This discovery has later led to the establishment of truly innovative targeted oncology HGF Receptor tyrosine kinase (RTK) Met drugs	a	0.22	0.22	Drug development or testing
BC	NANOMA (224594)	Nanorobotic delivery systems to improve the administration of drugs	h, AI	3.90	2.46	Drug development or testing
BC	DDRESPONSE (259893)	Olaparib/Lynparza as the first PARP inhibitor approved in Europe and the US in December 2014	a	8.17	6.00	Drug development or testing
BC	ONCOLYTIC-HERPES (340060)	Oncolytic herpes simplex viruses (oHSVs) targeted at cancer cells	a	2.48	2.48	Drug development or testing
BC	MERIT (601939)	BioNtech—The first vaccination with the highly individualized Mutanome RNAs, targeting patient-individual mutations	a	7.79	5.96	Drug development or testing
BC	BIOVALID (684862)	BIOVALID was boosted by the approval of palbociclib in EU in November 2016	h	0.68	0.68	Drug development or testing
BC	NoCanTher (685795)	Potential treatment leap—clinical trial granted authorisation at the end of project cycle	a, h, AI	7.11	7.11	Drug development or testing
BC	ONCOTHERANOSTICS (795272)	Advanced Theranostic Nanomedicines related to PD-L1 checkpoint inhibitor drugs	a	0.26	0.26	Drug development or testing
BC	SMARTRIOX (811744)	Potentially breakthrough discovery—Targeted nanoparticles-based drug delivery system development for BC	a, h	2.85	1.99	Drug development or testing
PC	RV001 (879817)	Potentially breakthrough discovery—RhoVac is developing the first prophylatic vaccine against metastasis	h	5.27	2.50	Drug development or testing
BC	RATHER (258967)	Phase Ib/II clinical trial of a novel kinase inhibitor drug called ‘taselisib’	h	7.81	6.00	Development of treatments or medical devices
BC	LIGHT2NANOGENE(624888)	Cell surgery via nanotechnology	h	0.28	0.28	Development of treatments or medical devices
AD	NEURAM (204053)	potentially breakthrough discovery—Commercialization opportunity mentioned	a	4.27	4.27	Development of treatments or medical devices
BC	INTHER (725151)	The world’s first device-based laser immunotherapy, imILTCLS	h	2.14	2.14	Development of treatments or medical devices
BC	BigMedilytics (780495)	Big Data sets for 11 million EU patients	AI	16.95	15.00	Development of treatments or medical devices
BC	B2B (801159)	Potentially breakthrough discovery	h, AI	3.80	3.80	Development of treatments or medical devices
BC	REGENERA (812002)	Development of polyurethanesther capable of inducing breast tissue regeneration after mastectomy	a	2.70	1.89	Development of treatments or medical devices & prevention measures
BC	Olfactomics Surgery (848682)	FDA breakthrough designation for the MEDICAL DEVICE was obtained	h, AI	2.65	1.85	Development of treatments or medical devices
BC	ENVIROGENOMARKER (S226756)	Development of Biomarkers of Environmental Pollutant Toxicity	h	4.86	3.50	Prevention measures
AD	CILMI (247620)	Computational Intelligence in Lifestyle Management Infrastructure	AI	0.21	0.21	Prevention measures
AD	VirtualBrain Cloud (826421)	Revolutionary cloud-based brain simulation platform to support personalized diagnostics and treatments in neurodegenerative diseases	AI	15.01	15.01	Development of treatments or medical devices & prevention measures

Source: authors’ elaboration based on data processing results

Where:

a—animal model

h—human model

AI—AI/in silico/computational models

**Table 13:**
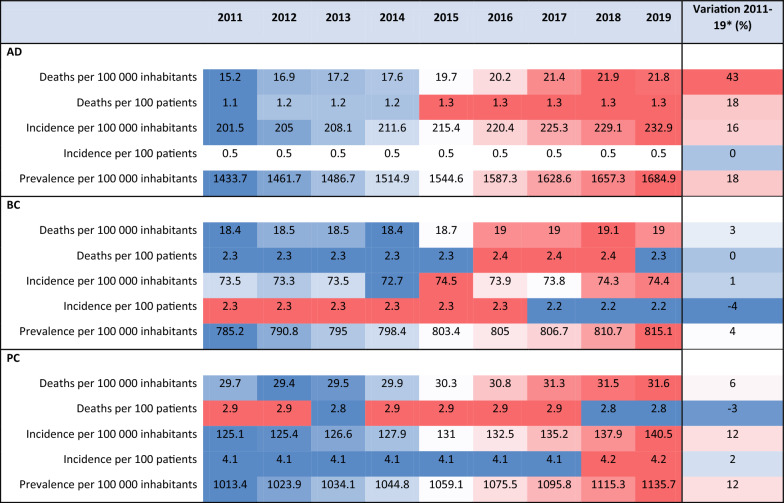
Major public health trends in AD, BC and PC across EU28 countries (indicator 12)

Where:

^*^ Variation = (Value2019–Value2011)*100

Prevalence = total number of cases in the population (see GHDX tool https://ghdx.healthdata.org/gbd-results-tool)

Incidence = number of new cases in the population (see GHDX tool https://ghdx.healthdata.org/gbd-results-tool)

Death = the permanent disappearance of all vital functions without possibility of resuscitation at any time after a live birth has taken place; this definition therefore excludes foetal deaths (stillbirths) (see Eurostat Statistics Explained Glossary https://ec.europa.eu/eurostat/statistics-explained/index.php?title=Glossary:Death)

Inhabitant = usual resident population (see Eurostat Statistics Explained Glossary https://ec.europa.eu/eurostat/statistics-explained/index.php?title=Glossary:Population_figure)

Patient = each individual registered/assigned to or regularly seen by a health provider (see WHO Glossary of Terms https://www.euro.who.int/__data/assets/pdf_file/0006/421944/Glossary-web-171219.pdf)

### Education, training and job opportunities

#### Staff/researchers

For the total number of staff/researchers, the information was only available for H2020 projects, through DG RTD’s final reporting summaries. The data for FP7 was incomplete, and covered only 41 projects out of the more than 200 projects analysed. 5,445 researchers were involved in the 198 projects assessed in the frame of the H2020 programme. The highest number of researchers was involved in the BC-related projects, which actually represented more than half of the H2020 projects assessed. This results in an average of 24 researchers involved in one project for BC. The average number of researchers was much higher for the AD-related projects, i.e. approximately 35 researchers per project. (See Table 14).

**Table 14:** Number of staff/researchers employed in H2020 (indicator 13)

	No of researchers	Animal models	Animal models (exclusive)	Other (non-animal) models	Human model (exclusive)	In-silico/AI model (exclusive)
AD	2.396	1.571	812	825	357	350
BC	2.605	1.147	83	1.458	521	706
PC	444	276	10	168	84	43
Total	5.445	2.994	905	2.451	962	1.099

Source: authors’ elaboration based on data processing results

#### Learning opportunities

A fully-fledged analysis of the learning opportunities resulting from the projects analysed was not possible due to the complexity of the data and the qualitative information that needed to be assessed. However, a qualitative analysis was undertaken as follows:

- For FP7 and H2020 projects, a selection of projects was made, based on the research programme ‘title’ and project topic name. The following were selected:
- Project topic name:
  - Marie-Curie Actions: ‘Intra-European Fellowships for career development’ (FP7)
  - Marie-Curie Actions: ‘Initial Training Networks’ (FP7)
- Programme title:
- Marie Sklodowska-Curie: ‘Innovative Training Networks’ (H2020)
  - Marie Sklodowska-Curie: ‘Innovative Training Networks’ (H2020)
  - Strategy for exploitation of research results funded under Euratom Research and training Programmes in the field of radiation protection
- For FP5 and FP6 a search by key work ‘training’ was performed on the project’s objective description.

A total of 54 projects were selected, based on the criteria described above. (See Table 15).

**Table 15:** New learning opportunities (indicator 14)

	No of projects	Animal models	Animal models (exclusive)	Other (non-animal) models	Human model (exclusive)	In-silico/AI model (exclusive)
AD	22	11	3	11	4	2
BC	23	12	3	11	7	2
PC	9	5	2	4	2	1
Total	54	28	8	26	13	5

Source: authors’ elaboration based on data processing results

The highest number of learning opportunities can be identified for BC biomedical research area (23), followed closely by AD (22). It is worth noting that the total volume and the EC contribution respectively for the AD area are remarkably higher than for the BC research area. The share of EC contribution for these projects was at 91% of their total costs. Nevertheless, overall these represent a small share of the total volume and EC contribution for all projects analysed (i.e. respective 6% of the total volume, and 7% of EC contribution).

## Discussion

AD remains an ongoing unresolved issue in all major world regions. This is particularly the case in rapidly aging industrialized societies of the northern hemisphere [40]. Most typically, wealthy OECD regions such as the North America, Japan [41] and rich Western Pacific nations and old EU-15 Western European [42] countries expose the most of vulnerability [43]. These societies are characterized by considerably stronger urban and rural networks of nursing facilities for the elderly and substantial ability to invest in home based medical care [44]. Yet their accelerating pace of population aging and shrinking workforce puts substantial pressure to extend retirement age policies [45]. This affects both those suffering from various dementias during their late life decades while still in the labour market and their family caregivers imposing substantial burden on entire families and households [46]. For these reasons, there is an increasing gap between the ability of these societies to devote higher health spending into advanced medical care and everyday assistance and disposable human workforce necessary to provide these services. Despite Robotic 4.0 revolution is ongoing, it is still far from provision of replacement for standard nursing care [47].

Unlike in the rich industrialized North, the developing nations of Global South faces different challenges [48]. Burden of dementia remains substantial in global terms due to respective population size yet far lower prevalence and incidence. Yet here the stronger side is actually the opposite. Universal health coverage is a distant dream in many of these countries across Sub-Saharan Africa, South Asia and Latin America [49]. The health expenditure projections until 2025 [50] and 2030 even for rapidly developing Emerging BRICS Markets remain stable and suggest strong real GDP growth [51]. Yet the spending share devoted for the elderly is far less significant compared to OECD average. Furthermore, this is the case even for China which will take over the lead from Japan as the fastest aging large nation as we approach 2050 [52]. There are huge capacities to support rise of welfare society of the BRICS and other emerging economies including Indonesia, Mexico, Nigeria and Turkey. Yet despite this fact, their priorities for public spending particularly governmental share are significantly different compared to the West. Notable example are wealthy Arab Gulf countries which spend around 3% of their GDP on health care unlike 9–10% common for even middle-income European countries [53]. These facts make establishment of network of supportive facilities for elderly suffering from dementias slow and insufficient with notable drug shortages and deficits [54]. Yet on the side of the strength family caregiving is still quite traditional and strong in most Asian and African countries. Certain Israeli findings claim that even up to two million people in a country of eight, take care of a close family member, elderly parents, spouse or a child [55]. Even under the condition of below replacement fertility levels in mainland China, South-East Asian ASEAN societies family care for elderly suffering from dementia remains widespread phenomenon [56].

Clinical oncology landscape of breast cancer and prostate cancer exposes far less peculiar differences among wealthy and LMICs societies. Morbidity burden of BC and PC unfortunately continues to grow across the Global South although it has reached certain plateau epidemiology in Europe and elsewhere [57]. Notably, the investment to achievement ratio particularly in BC research looks far more beneficial compared to AD. As previously noted in the findings of this study, several dozens of truly innovative pharmaceuticals have been patented and penetrated the US, Japanese, Canadian, EU5, South Korean, Taiwanese and other high-income markets during the past two decades [58]. Many of these treatment algorithms alongside with far more sensitive imaging diagnostics, biopsies and tumour markers, have made survival chances far better [59]. Extended life prognosis and improved quality are common to many histological sub-entities of these two cancers and variety of their clinical stages upon diagnosis [60]. Thus, the fruits of investment in scientific and technological breakthroughs in these clinical entities remain substantial in all three major ICH regions of North America, Japan and EU [61].

Over the course of the first two decades of the twenty-first century, the EC has been steadily increasing both the volume of projects being funded and the amounts of financial resources being allocated on AD, BC and PC research [62]. This trend has continued throughout four consecutive science funding cycles, namely: FP5, FP6, FP7 and H2020. Notably, public–private partnerships in terms of engagement of large industry together with universities-based consortia have led to some of the most impactful projects being funded. In order to track the overall success rates of these large science financing streams, the JRC and GOPA worked together on an array of 14 plausible indicators.

Some important considerations can be derived from our retrospective assessment of EU research funding on these selected biomedical research areas. In particular, figures derived from ‘Funding and Economic’ and ‘Scientific and Technological’ indicators globally suggest that despite more resources were allocated on AD and dementia than BC and PC research over the last two decades, translational success in drug development for AD has been considerably lower (e.g., in term of new drug development) than for oncology, which is in line with recent analyses [63].

Noteworthy, 23% of the analysed AD projects made exclusive use of animals (entire living animals or animal-derived materials), whilst this percentage was lower for BC- (15%) and PC-related projects (13%). Designing research proposals exclusively on the use of animals, over-reliance on animal models of human diseases, and general lack of knowledge on human-relevant approaches or new approach methodologies (NAMs), spanning sophisticated human 3D cell culture models, organoids, organ-on-chip technologies and microfluidics, computational modelling, neuroimaging technologies, to name a few, may all contribute to translational failure of basic and applied biomedical research (Pistollato F et al. Animals (Basel). 2020 Jul 14;10(7):1194.) (Pound et al. J Transl Med. 2018 Nov 7;16(1):304.).

When looking at the ‘scientific and technological’ indicators, the number of registered and filed patents generally increased during the most recent FPs (FP7 and H2020), and was higher in BC research compared to AD and PC research. A higher translatability of BC research may be reflected also by this higher number of patents. Additionally, 76% of these BC-related patents (70 in 92) originated from research that was conducted using a variety of different methodological approaches. As a similar trend, most of novel diagnostic tools were developed in the context of research projects based on non-animal methods (i.e., in silico/AI) (in total, 86 in 102 projects).

Across FP5, FP6, FP7 and H2020, 64 projects targeted the development, testing or repurposing of drugs, with the great majority of these targeting drug development. However, establishing a causal connection between a certain EU-funded grant and a consecutive new drug launch is a challenging task. This is mainly because the synthesis and development of a new active ingredient, with a novel molecular mechanism of action, remains exceptionally expensive and requires a lengthy time investment, with only one or two in 10,000 tested drugs making it through to become licensed treatments [64].

Notably, by assessing the number of clinical trials across the analysed FPs and the selected disease areas, more clinical trials were conducted over time on BC (which tripled over time) than for AD or PC. This could be considered as a further reflection of the higher translational impact of BC research, at least in the context of this retrospective analysis.

**Fig. 4:**
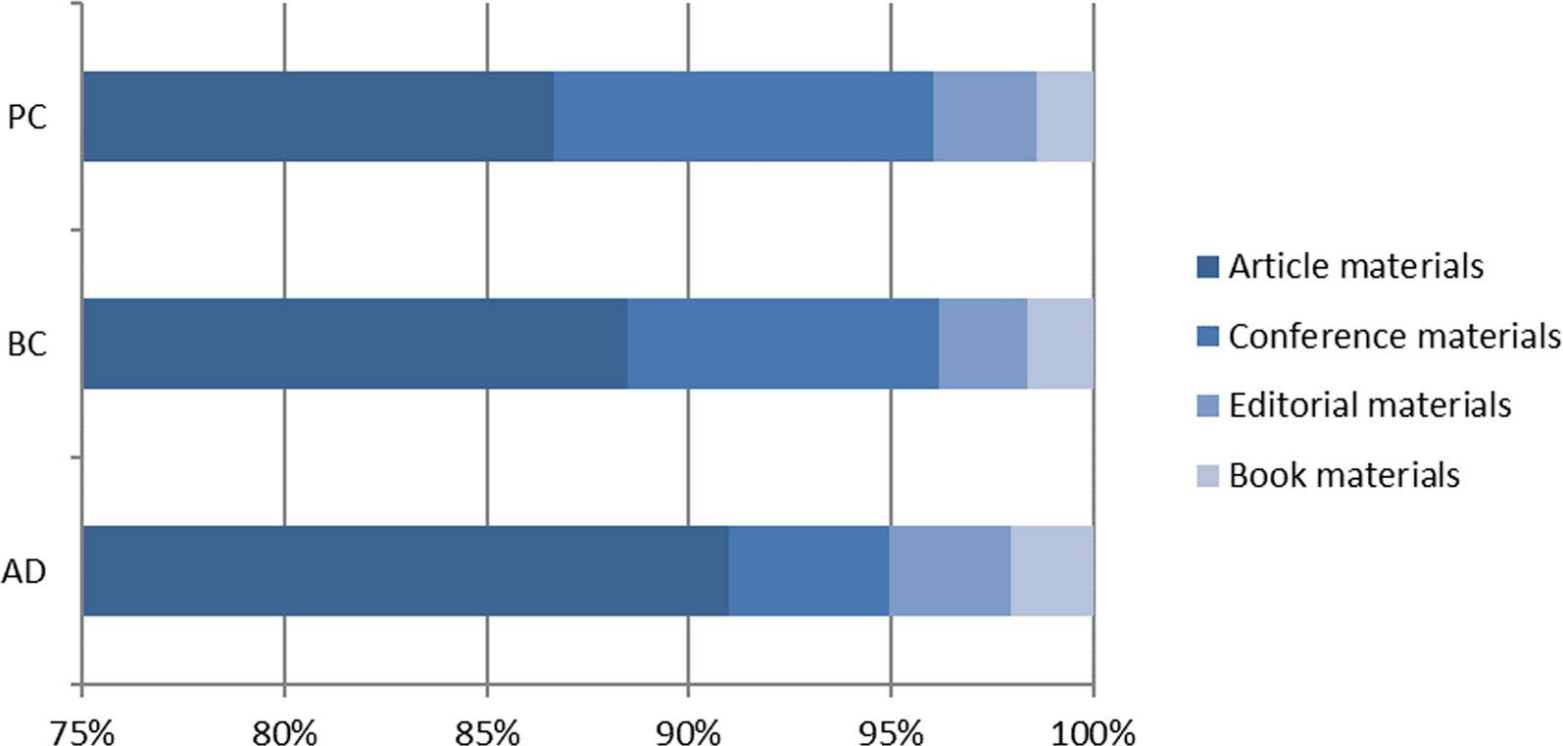
Share of publications by type and biomedical research area

**Fig. 5:**
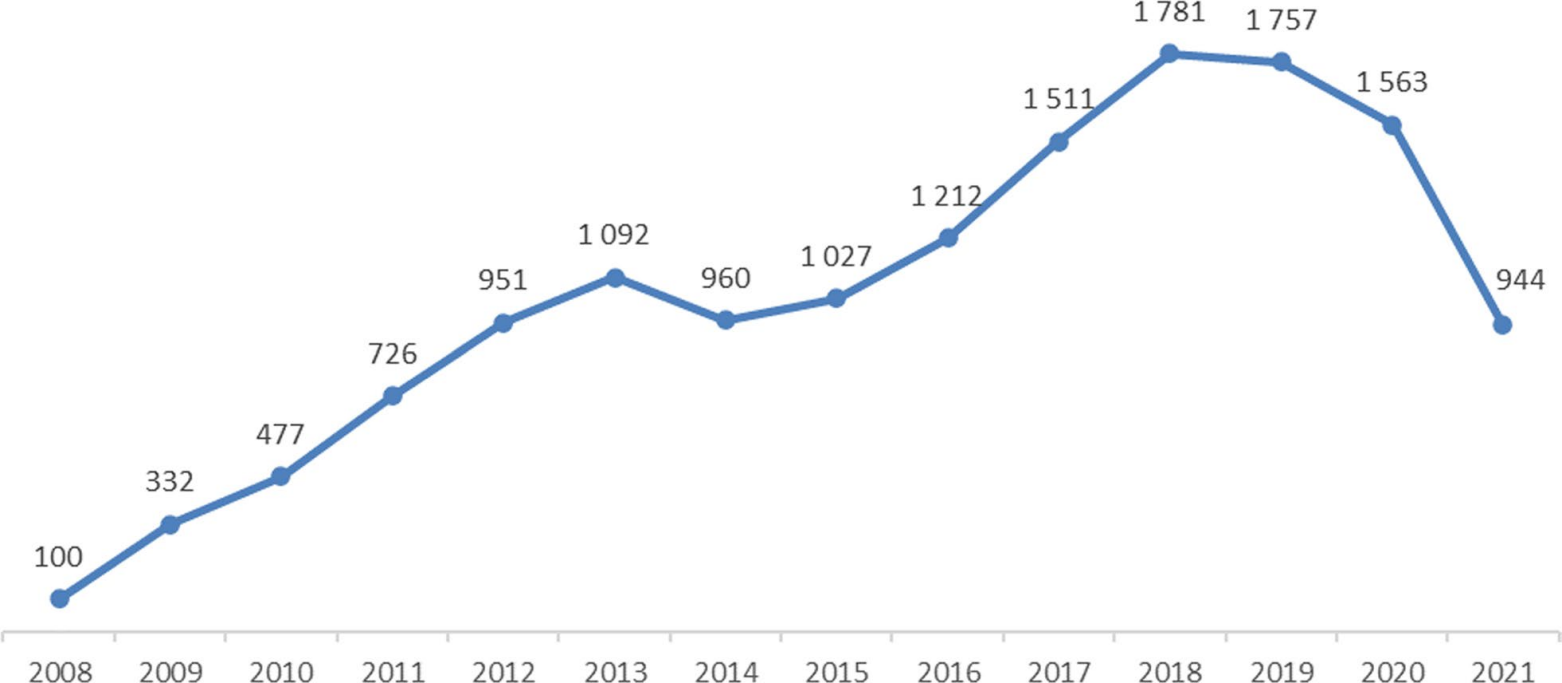
Number of publications by year of publication, 2008–2021

By looking at the ‘dissemination’ indicators, large extent publications output, its global visibility and readership amongst its targeted audience and cumulative citations grew tremendously over the 1999–2019 time period. (Please see Figs. 4 and 5.) The number of publications derived from AD projects was the highest, followed by those on BC research. While the number of publications generally reflects the contribution of research to generate scientific knowledge, this may not be directly proportional to the level of translational success of such knowledge. Especially in recent years, the realization of scientific findings in device or treatment development has significantly lagged behind; this may also be linked to the fact that scientists have progressively allocated their time and resources on publication-generating work (rather than translational research) to maintain funding and professional advancement, as commented by Fernandez-Moure [65]. Additionally, as the ‘citation’ indicator could not distinguish self-citations, the measurement of the genuine reflection of scientific influence of any published study could not be performed in the context of this activity. While excessive self-citation can be a problem, there is also no clear consensus on how much is too much or on what to do about the issue [66, 67].

By looking at the ‘regulatory and policy’-related indicators, across the analysed FPs, in total 45 projects targeted prevention measures for the three diseases, most of them (26 in 45) were on BC research and were explored in most recent FP (i.e., H2020). Investment in prevention is crucial to reduce the burden of NCDs, with several primary prevention, life-style interventions, spanning physical exercise, healthy diet, weight reduction, quitting smoking and alcohol and improvement of sleep quality, suitable to reduce the risk of BC [68], PC [69] and AD [70]. Investing in prevention could help reduce the mortality and incidence of these NCDs. In line with this, our retrospective assessment of ‘major public health trends’ over the period 2011–2019 showed that the incidence (per 100,000 inhabitants) of BC increased only by 1% compared to AD (16%) and PC (12%). This could be linked to the higher level of investment in preventive measures in this area of biomedical research. This is also in line with previous analysis, indicating that prevention, as well as early detection and early treatment of common cancers [71], as well as AD [72], would have a major economic benefit worldwide, reducing incidence and mortality.

Finally, EU funding allocated on these biomedical research areas represented an incredible opportunity for young scientists to acquire new knowledge and expertise. The ‘education/training’ and ‘learning opportunities’ indicators showed that a higher number of researchers was involved in BC-related projects. Noteworthy, despite the total volume and the EC contribution for the AD area were remarkably higher than for BC, the number of learning opportunities between these two research fields was found to be similar (23 for BC and 22 for AD).

## Conclusive remarks

As AD, BC and PC continue to hold a high place in the EC’s research and innovation agenda[11], a toolbox of indicators may play key role to provide insights on the outputs and impact of research in these biomedical areas. Concerning the selected indicators, on the input side of the equation, probably, the most significant indicators primarily refer to those ones related to the scale of EC’s financial involvement and contributions of national and overseas donor agencies. On the side of output, the most relevant are those indicators exposing scientometric outputs in terms of publications, citations and social networks attention and ultimately novel drug marketing authorisations and patents [73].

The sensitivity and specificity of these indicators might be improved with the plausible involvement of AI algorithms and real-time tracking. Most of the perceived significance of the concluded grants shows up a year or two after the funding expiration. In addition, large segments of FP5 and FP6 outputs belonging to the era preceding modern electronic publishing, being hardly accessible for retrospective monitoring. Increasing our capability to seek both deeper into the past and further into the future might substantially change our ability to perceive the overall outreach of science funding.

It is important to emphasise that targeted oncology pharmaceuticals, small molecules and monoclonal antibodies alike, have been the most prominent source of innovation in BC and PC research, extending patients’ survival and increasing their life quality. Unlike oncology, dementia drug development was way less successful with only minor improvements related to the quality of supportive medical care for symptoms and earlier, more sensitive diagnostics without any disease-modifying treatment. Due to these funding streams, a large scale capacity building of professional networks in these areas took place across Europe, increasing the chances for mutual fertilisation of ideas.

Yet, truly breakthrough discoveries remain scarce, with some notable exceptions in imaging diagnostics and nanotechnology. These prominent cases of essential innovation remain largely driven by participation of medical device industry multinational companies. EU-funded clinical trials took place leading to the development of novel drugs, featuring novel mechanisms of action. This fact refers to personalised medicines interfering with metastatic processes in oncology and tumour growth. There are indeed some truly success stories being prime examples of outputs of European investment for science. These prominent cases of breakthrough discoveries serve as an evidence of the European capability to generate cutting-edge technological innovation in biomedicine in the global arena [74].

In conclusion, on the basis of our analysis, some considerations could be proposed to possibly prioritize research strategies in biomedical sciences. In particular, research projects that focus on multidisciplinary, human-relevant approaches, tackling prevention, early diagnosis, epidemiology, drug/treatment development and with clinical settings seem to be more conducive to translational success and social impact. In line with this consideration, current Horizon Europe funding scheme aims to support epidemiology and computational modelling especially to respond to infectious disease outbreaks, as the most recent COVID19 [75]. While this is relevant for communicable/infectious diseases, it is also highly relevant for NCDs, like cancer and dementia, as also underlined in a recent JRC survey addressed to EU-funded researchers in these fields of biomedical science [76]. Ultimately, some less efficient fiscal flows in poorly productive areas of research could be reconsidered as priorities when shaping the agenda of future science funding programmes.

## Data Availability

The data supporting the results reported in this paper can be accessed freely from the official portals of the European Commission such as EuroStat, DIrectorate General published reports, Joitn Research Centres publications and data repositoria. This includes both primary data and secondary data used in this study.
